# Breaking Patterns: A Case of Dieulafoy Lesion in a Young Patient Without Comorbid Conditions

**DOI:** 10.7759/cureus.60513

**Published:** 2024-05-17

**Authors:** Melissa A Postoll-Downs, Fatma Ozguc, Jaya Vasudevan, Peter M Stawinski, Randy Wright

**Affiliations:** 1 Internal Medicine, San Antonio Uniformed Services Health Education Consortium, San Antonio, USA; 2 Internal Medicine, The University of Texas Health Science Center at San Antonio, San Antonio, USA; 3 Gastroenterology, The University of Texas Health Science Center at San Antonio, San Antonio, USA

**Keywords:** hematemesis, upper gastrointestinal bleed, endoscopy, splenic artery embolization, dieulafoy lesion

## Abstract

A Dieulafoy lesion is an abnormal artery located in the gastric submucosa that represents a rare cause of upper gastrointestinal bleeding. These lesions typically present as massive hemorrhages in older patients, with multiple medical comorbidities. The lesions are diagnosed with endoscopy and treated with hemostasis by clip placement or coagulation. This case report is that of a rare presentation of this rare condition in a younger 18-year-old patient with no medical comorbidities. He presented with hematemesis, melena, and syncope in the setting of ibuprofen self-treatment for a recent upper viral illness. This medication use is a proposed inciting factor for the bleeding lesion, though he had a history of a splenic artery embolization following a remote motor vehicle accident, which could represent a mechanism for a rare acquired lesion. A gastroenterologist was consulted and assisted in the diagnosis and management of this patient. His lesion was identified and treated within 24 hours of his presentation.

## Introduction

A Dieulafoy lesion is an abnormal, tortuous, large-diameter, non-tapering artery located in the gastric submucosa that may protrude through a small mucosal defect [1]. These lesions represent a rare cause of upper gastrointestinal (GI) bleeding, accounting for approximately 1-2% of all acute upper GI bleeds, though they can even more rarely occur in the lower GI tract [1,2]. The mortality of these lesions has decreased from above 80% to approximately 9-13%, with the advent of more advanced endoscopic hemostasis techniques [2,3]. The clinical presentation is typically recurrent, massive hemorrhage in the form of hematemesis or melena. Ninety percent of patients have multiple medical comorbidities, with a 2:1 male-to-female predominance, and the age at presentation is typically above 50 years [1,2]. The most common associated comorbid conditions include cardiovascular disease, hypertension, chronic kidney disease, diabetes, and alcohol use disorder [4].

Dieulafoy lesions are usually diagnosed during endoscopy, and the diagnostic yield of the procedure is the highest during an acute, active bleed [5]. If endoscopic methods fail to locate the lesion, computed tomography (CT) angiography is useful, especially for lesions in the colon or rectum. The endoscopic hemostasis is achieved with clip placement or coagulation [6]. In cases of re-bleeding episodes, repeat endoscopy is reasonable, though angiographic embolization or surgical resection may be considered.

## Case presentation

This is a case of an 18-year-old male patient with a history of splenic artery embolization following a motor vehicle collision two years prior to presentation, who presented to the emergency department after an episode of large-volume hematemesis associated with syncope and melena. One day prior to developing these symptoms, he had finished an approximately four-day course of over-the-counter ibuprofen at manufacturer-recommended doses (400-600 mg every six to eight hours) for a recent upper respiratory infection. He had previously consumed alcohol, with a reported history of one to two drinks once per week, but had not consumed any alcohol in at least two months. He denied any prior tobacco product use, *Helicobacter pylori *infections, peptic ulcer disease, and use of antiplatelet agents, including aspirin. He had no history of anemia, with prior laboratory tests indicating a baseline hemoglobin of 12.7-13.1 g/dL. 

His blood pressure on arrival at the emergency department was 88/59 mmHg, pulse was 78 bpm, respiration rate 16 breaths per minute (brpm), 100% oxygen saturation on room air, and he was afebrile (36.7°C). Initial laboratory values were notable for hemoglobin 8.7 g/dL, with a mean corpuscular volume of 88.8 femtoliters (fL) and a mean corpuscular hemoglobin concentration of 33.3 g/dL. International normalized ratio (INR) was 1.4, blood urea nitrogen was 39 mg/dL, and lactic acid was 3.1 mmol/L. Given his history of large-volume hematemesis, two units of packed red blood cells were empirically transfused with an appropriate hemoglobin and blood pressure response to 10.2 g/dL and 103/63 mmHg, respectively. He was then treated with high-dose intravenous proton pump inhibitor therapy.

**Figure 1:**
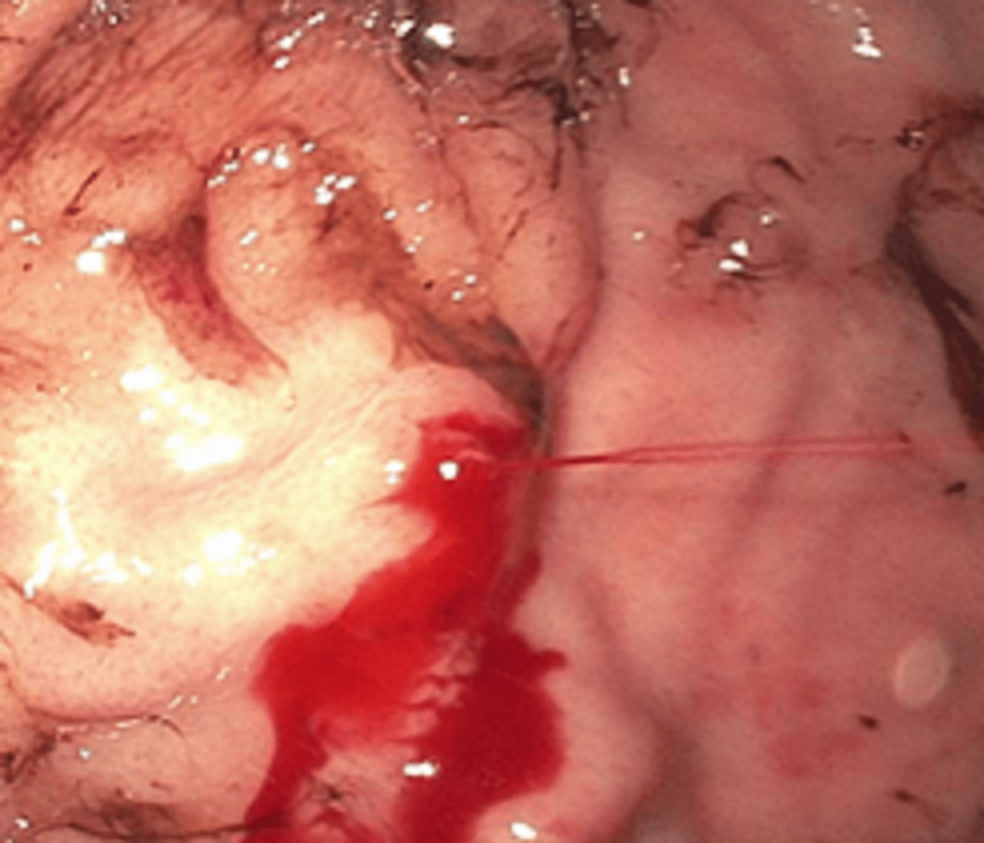
Active spurting of the Dieulafoy lesion

**Figure 2:**
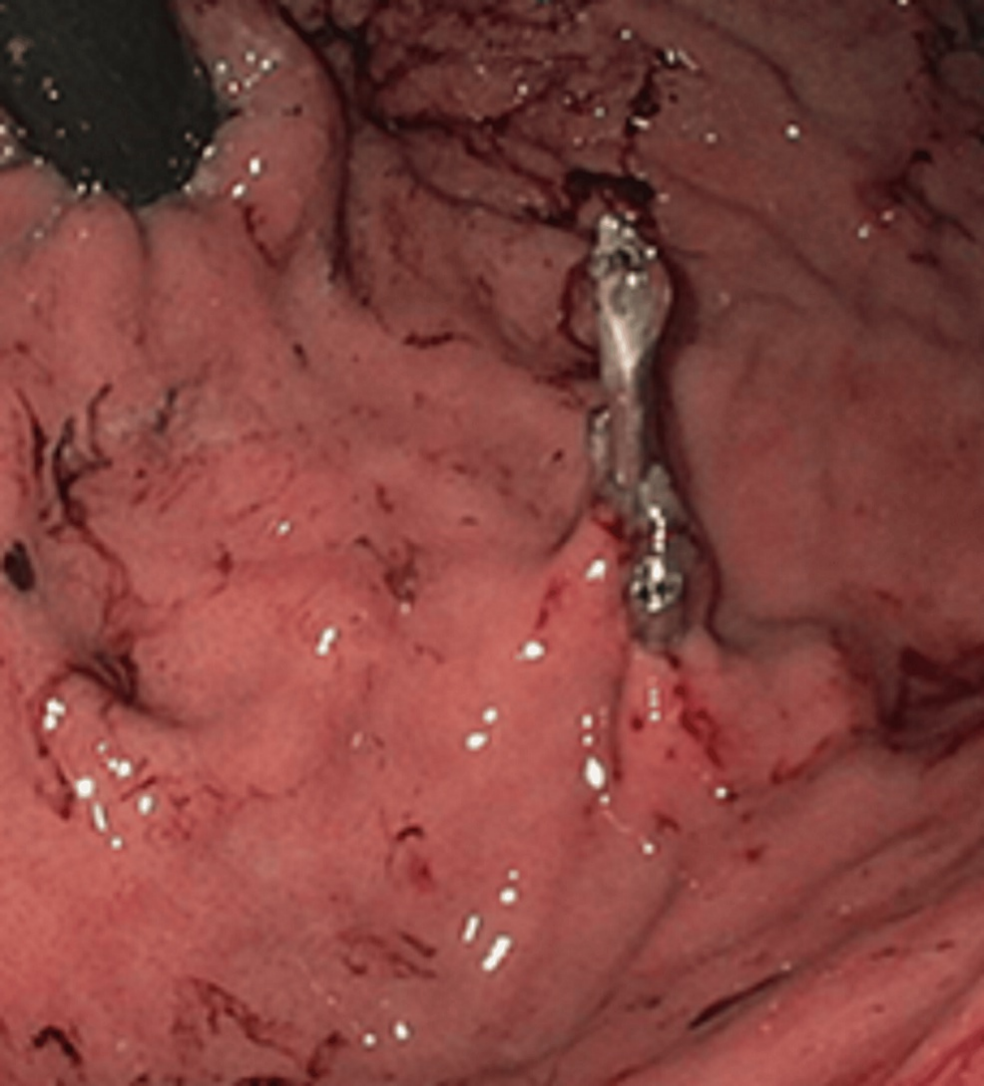
Hemostasis achieved with the placement of three mechanical clips

A gastroenterologist was consulted, and an urgent esophagogastroduodenoscopy (EGD) was performed within 24 hours. An actively spurting, aberrant arterial lesion with stigmata of recent bleeding, was found in the gastric fundus (Figure 1), with fresh blood visualized in the gastric body. Three hemostatic clips were successfully placed to achieve hemostasis without any further complications (Figure 2). The patient was observed for development of re-bleeding or other complications for an additional 24 hours and was discharged home with instructions to avoid alcohol and all non-steroidal anti-inflammatory medications, likely indefinitely. He was given strict return precautions regarding signs or symptoms of upper and lower GI bleeds. On follow-up, four months later, there was no evidence of recurrent bleeding episodes. 

## Discussion

The etiology of Dieulafoy lesions remains poorly understood. The detection of Dieulafoy lesions in neonates suggests a congenital pathology [7]. It is widely believed that the majority of these lesions are congenital, with presentations later in life due to erosion through the submucosa and repetitive damage from pulsatile flow through the dilated artery. While there is a potential association between using nonsteroidal anti-inflammatory drugs (NSAIDs) and alcohol, which could contribute to local mucosal injury and reveal abnormal vessels, more data are required to validate this hypothesis [1]. Cardiovascular disease is commonly identified as a risk factor for bleeding from Dieulafoy lesions; however, this association has not been studied given the rarity of these lesions. Cardiovascular disease induces vascular injury through structural changes and inflammatory processes, thereby theoretically increasing the likelihood of complications such as bleeding from these lesions.

Limited cases of acquired Dieulafoy lesions are documented in the literature but are recognized to be a possible sequela of arterial trauma. There have been reports of Dieulafoy-like lesions in the bronchial arteries in individuals with a history of chronic smoking [8]. Another gastric Dieulafoy-like lesion has been reported, secondary to an abnormal blood supply from the left phrenic artery diverted to an enlarged splenule, in an asplenic patient [9]. Additionally, Faraji et al. reported the formation of Dieulafoy-like, hypertrophied collateral arterial lesions in a patient with concurrent splenic vein and artery thrombosis [10]. The noteworthy aspect of this case is the occurrence of a Dieulafoy lesion in a young patient without comorbidities. The pathophysiology of this lesion epidemiologically as well as in this case is unknown. There are proposed mechanisms for both inherited and acquired [3]. The lesion, in this case, was plausibly precipitated by recent ibuprofen usage for a recent viral illness. He had previously consumed alcohol, but not in a reported timeline that would have plausibly contributed to the presentation. Considering the patient's history of splenic artery embolization, this raises the possibility of an acquired lesion.

The typical location for these lesions is in the stomach along the high lesser curvature, near the gastroesophageal junction, though they can be seen throughout the GI tract. In the absence of active bleeding, the Dieulafoy lesions are difficult to visualize as they may appear as a small and subtle raised area without an associated ulcer. The primary approach for managing bleeding in a Dieulafoy lesion involves endoscopic treatment. This can include a combination of therapies, such as injections of epinephrine followed by thermal coagulation, or placement of hemostatic clips directly over the lesion. The risk of recurrent bleeding after endoscopic treatment is relatively high at 10% in the first seven days [3], primarily attributed to the substantial size of the underlying artery. For this reason, the site of a Dieulafoy lesion is usually tattooed for future reference, and for patients experiencing recurrent bleeding despite endoscopic treatment, surgical resection, or interventional radiology (IR)-guided arterial embolization is recommended [11].

## Conclusions

In conclusion, a Dieulafoy lesion is a rare condition that can result in severe upper GI bleeding. Diagnosis, even in typical presentations, can be challenging due to intermittent episodes of bleeding. Early diagnosis of the lesion is imperative and is accomplished by endoscopy or CT angiography. Treatment with hemostasis within 24 hours can prevent life-threatening complications, though recurrence of bleeding can occur. Keeping a broad differential, with consideration for Dieulafoy lesions in younger patients without comorbidities but plausible mechanisms for acquisition or inciting factors for a bleed, can help reduce mortality in this more atypical population.
